# Use of Pharmacologic Agents for Modulation of Ischaemia-Reperfusion Injury after Hepatectomy: A Questionnaire Study of the LiverMetSurvey International Registry of Hepatic Surgery Units

**DOI:** 10.1155/2014/437159

**Published:** 2014-11-12

**Authors:** Santhalingam Jegatheeswaran, Saurabh Jamdar, Thomas Satyadas, Aali J. Sheen, Rene Adam, Ajith K. Siriwardena

**Affiliations:** ^1^Hepato-Pancreato-Biliary Surgery Unit, Manchester Royal Infirmary, Manchester M13 9WL, UK; ^2^Centre Hépato-Biliare, Hôpital Paul-Brousse, 12 Avenue Paul Vaillant Couturier, 94800 Villejuif, France

## Abstract

*Objectives*. This study is a questionnaire survey on the use of pharmacological agents to modify liver ischaemia-reperfusion (IR) injury in patients undergoing hepatectomy for colorectal liver metastases with the target population being those units participating in the LiverMetSurvey international registry. *Methods*. Members of LiverMetSurvey were sent an online questionnaire using SurveyMonkey comprising ten questions on the use of pharmacological agents to modulate hepatic IR injury in the perioperative period after hepatectomy. The questionnaire was sent to 446 clinicians registered with the LiverMetSurvey. There were 83 (19%) respondents. *Results*. Fifty-two (77% of 68 respondents to this question) never used pharmacological agents to modify liver IR injury during hepatectomy. Thirteen (19%) used pharmacological agents selectively. Three (4%) used these routinely. N-Acetylcysteine was the most widely used pharmacological agent with equal distribution of use around intraoperative and postoperative periods. *Conclusions*. This is believed to be the first survey on the use of pharmacological agents to modify liver IR injury. The target population is clinicians involved in liver resection. The results show that pharmacological modulation is used by only a minority of respondents to this questionnaire and that when this treatment is selected, N-acetylcysteine is the most frequently used.

## 1. Introduction

Hepatectomy is established as a standard of care for patients with resectable liver-limited metastases of colorectal cancer origin [1, 2]. Hepatectomy is also undertaken for a range of other malignant and benign conditions [3, 4]. Contemporary experience with techniques such as downsizing of tumours by neoadjuvant chemotherapy [5] and modification of the volume of the future remnant liver by selective portal vein embolization [6] results in the ability to offer liver resection to a greater proportion of patients with liver metastases. These changes, coupled with population trends resulting in a greater proportion of elderly patients presenting for treatment [7], mean that, in the 21st century, liver resection for metastatic colorectal cancer is often undertaken in older individuals and/or in patients whose hepatic functional reserve may have been compromised by prior chemotherapy or by preexisting chronic liver disease [8]. Although reports outline the feasibility of undertaking liver resection with low perioperative mortality [9, 10], postoperative morbidity is important as it can influence a range of outcomes including critical care occupancy and in-patient stay, uptake of adjuvant chemotherapy, and quality of life after surgery.

During liver resection, IR injury can occur during any of a series of operative steps including liver mobilisation and permanent inflow occlusion of the segment to be resected and during temporary inflow occlusion followed by restoration of blood flow to the future remnant liver (the Pringle manoeuvre). IR injury is an important determinant of postoperative morbidity and eventual clinical outcome [11, 12]. Strategies employed to minimise IR injury include avoidance of inflow occlusion and ischaemic preconditioning [13] and the administration of pharmacologic agents [14].

At the cellular level, Kupffer cells release proinflammatory mediators during liver IR, including oxygen-derived free radicals [15]. The physiologic intracellular defence mechanisms involved in the regulation of oxidative injury include enzymatic pathways catalysed by superoxide dismutase and glutathione peroxidase [16]. Glutathione (GSH), a tripeptide composed of glycine, glutamic acid, and cysteine, constitutes the largest component of the endogenous thiol buffer for oxygen-derived free radicals [17]. For tissue GSH synthesis, the availability of cysteine is generally the limiting factor [17].

An important exogenous cysteine precursor is N-acetylcysteine (NAC) [18]. N-Acetylcysteine is a thiol containing synthetic compound [18]. In the isolated, perfused rat liver, administration of NAC before reperfusion results in concentration-dependent increases in GSH concentrations in bile with corresponding increases in bile flow [19, 20]. Currently the most established clinical use for N-acetylcysteine is in the treatment of acetaminophen (paracetamol) poisoning [21, 22]. To date one small clinical trial has assessed the effect of perioperative n-acetylcysteine on prevention of ischemia-reperfusion injury in liver resection [23]. The dose used was similar to that used in the treatment of paracetamol overdose. Liver function was assessed by postoperative transaminase (ALT) rise and this was significantly ameliorated in the treatment group [23].

Despite the predominantly favourable results of experimental studies there are no substantive clinical trials evaluating the role of n-acetylcysteine or other pharmacological agents to modulate IR injury in patients undergoing hepatectomy. These drugs (in particular n-acetylcysteine) have been available for a long time and anecdotal evidence suggests that they are still used in an off-licence mode as modifiers of liver IR in hepatectomy. A questionnaire survey provides an overview of the contemporary use of pharmacological modulation of IR injury in liver surgical practice. An international target population of clinical units undertaking liver surgery can be obtained through the LiverMetSurvey.

Thus the aim of this study is to undertake a questionnaire survey of the use of pharmacological agents to modify liver IR injury in patients undergoing hepatectomy for colorectal liver metastases in units participating in the LiverMetSurvey.

## 2. Methods

### 2.1. Questionnaire Design

The questionnaire design examined strategies for modulation of liver IR injury during hepatectomy and included questions on a broad overview of techniques for this purpose in addition to pharmacological interventions. Specific detail was sought on whether pharmacological agents were used by respondents to treat or reduce liver IR injury with information being sought on the indications for use, choice of agent, use of a loading dose, timing of use in relation to the stage of surgery (preoperative, intraoperative, posttransection, etc.), and dose. As it was anticipated from prior literature searches that n-acetylcysteine would be the most widely used agent, specific focus was given to this drug [24, 25]. A final question sought opinion on whether the respondent supported in concept a randomized trial to evaluate the role of pharmacological IR modifiers in hepatectomy. The questionnaire is seen in the Appendix.

### 2.2. Target Population

Permission was obtained from the LiverMetSurvey for their members to be sent an online questionnaire using SurveyMonkey (https://www.surveymonkey.com/). The LiverMetSurvey was selected as this is a registry of liver resection outcomes for colorectal hepatic metastases and thus all participants would be likely to be undertaking hepatectomy. The sample size was all 446 participants in LiverMetSurvey. Response to this questionnaire was discretionary and following the initial email no reminders were sent.

### 2.3. Survey Details

The questionnaire was live for a period of six weeks from June 18, 2013, and was sent to 446 participants of the LiverMetSurvey. The survey link was emailed to potential participants by LiverMetSurvey. There were 85 respondents of whom two were not actively engaged in liver resection practice. These two respondents were excluded to provide a final study population of 83 (19%) respondents.

### 2.4. Ethics

The study was discussed with the hospital's research and development team, who advised that, as there was no patient contact and only email contact with professional colleagues, no ethics committee submission was required. Separate specific approval was obtained from LiverMetSurvey prior to sending the questionnaire.

## 3. Results

Not all respondents answered every question. The number of respondents to each individual question is provided and serves as the denominator for the percentage of respondents to that particular question. For several questions, more than one response was permitted and therefore the cumulative total of percentage responses to such questions can exceed 100.

### 3.1. Strategies to Minimise Liver IR Injury during Resection

**Figure 1:**
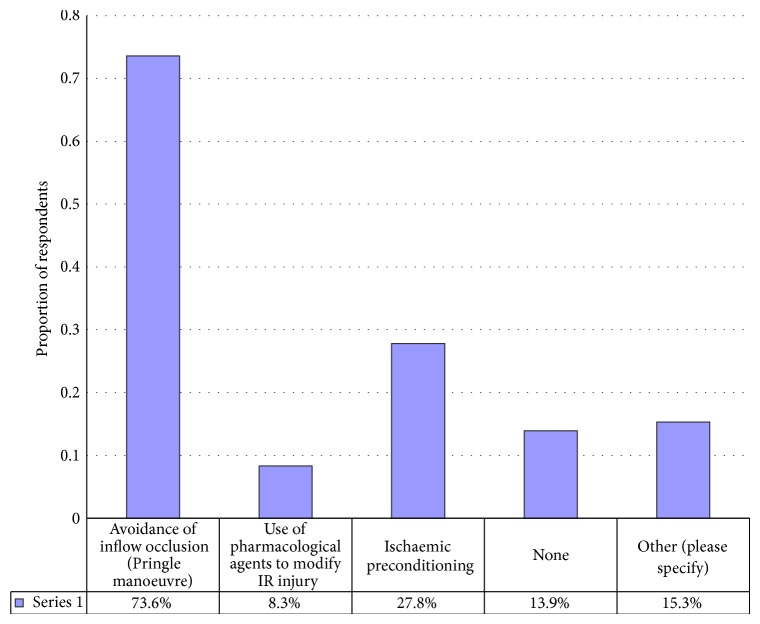
Strategies used to modify liver IR injury during liver resection (more than one response was permitted). There were 72 responses to this question. Other responses included use of sevoflurane anaesthesia, cooling of the patient's core body temperature, antioxidants, and probiotics in the preoperative period.

There were 72 respondents to this question and more than one response was permitted (Figure 1). The most frequently used strategy was avoidance of the Pringle manoeuvre by 53 (74%) respondents. Six (8%) replied that they used pharmacological agents to modify liver IR injury. Other individual responses are seen in Figure 1.

### 3.2. Use of Pharmacological Agents to Modify Liver IR Injury during Liver Resection

There were 68 respondents to this question. Fifty-two (77%) stated that they never used pharmacological agents to modify liver IR injury during hepatectomy. Thirteen (19%) used pharmacological agents selectively. Three (4%) used these pharmacological agents routinely.

### 3.3. Clinical Indications for Use of Pharmacological Agents to Modify Liver IR Injury

There were 18 respondents to this question. Ten (56%) stated that a requirement for total vascular exclusion was an indication for the use of pharmacologic agents, 9 (50%) stated that prolonged inflow occlusion was an indication, 9 (50%) used pharmacological agents if there was evidence of deterioration in liver function in the postoperative period, and 8 (44%) used pharmacological agents in the setting of a small-volume future remnant liver, 7 (39%) in major liver resection of more than 3 Couinaud segments, 5 (28%) in liver resections undertaken in patients with cirrhosis, and 4 (22%) in patients who had received prior chemotherapy.

### 3.4. Choice of Pharmacological Agent for Modulation of Liver IR Injury

There were 18 responses to this question. The only options provided for answers were n-acetylcysteine, glutamine, or “other.” Eight (44%) used n-acetylcysteine. Four (22%) used steroids. Individual replies were received (one respondent each) for glutamine, desflurane, sevoflurane, bicarbonate, arginine, and a cocktail of methionine with vitamins C and E.

### 3.5. Use of a Loading Dose of Pharmacological Agent to Modulate Liver IR Injury

There were 22 responses to this question with 14 (64%) replying that they did not use a loading dose.

### 3.6. Stage of Procedure for Use of Pharmacologic IR Modulation

There were 18 respondents to this question. Three (17%) used pharmacological intervention prior to surgery in the form of steroids. Timing in relation to surgery was not further clarified. Two (11%) gave NAC at induction of anaesthesia, four (22%) during liver mobilisation, two (11%) at commencement of transection, two (11%) postoperatively (routinely), and five (28%) postoperatively (selectively).

### 3.7. Use of N-Acetylcysteine as a Body Weight-Adjusted Dose or as a Standard Dose

There were 9 respondents to this from those who used n-acetylcysteine for modulation of liver IR injury. Seven (78%) used a standard dose for all patients and 2 (22%) used a body weight-adjusted dose.

### 3.8. Duration of Use of N-Acetylcysteine

There were 9 respondents to this question. Three (33%) continued the infusion for 48 hours, 3 (33%) until serum transaminases started to fall, 2 (22%) for 72 hours, and 1 (11%) for 24 hours. None continued until normalisation of serum transaminases.

### 3.9. Weight of Clinical Opinion for a Randomised Trial on the Use of N-Acetylcysteine to Modulate Liver IR in Hepatectomy

There were 69 respondents to this question. Fifty-six (81%) answered affirmatively and 6 (9%) replied that there should not be a trial. Reasons for a negative response were not sought. Seven offered no opinion.

## 4. Discussion

This study reports the results of what is thought to be the first survey of the use of pharmacological agents to modify liver IR injury in the perioperative period. Three important points must be emphasised when considering these findings. First, the results do not in any way represent an official position statement of the LiverMetSurvey. Second, given that less than 20% of the target audience responded to this questionnaire, the replies are not indicative of any consensus view. Rather, these are the responses of a minority. Third, it is possible that those who responded had a vested interest in pharmacological modification of liver IR injury and thus the results could be skewed by ascertainment bias.

The target population was selected as the LiverMetSurvey participants as by definition these are units undertaking liver surgery for colorectal hepatic metastases. Clearly, the views of units and clinicians outwith LiverMetSurvey will not have been captured by this survey.

Interpretation of these results bearing these caveats in mind produces an interesting overview into pharmacological modification of liver IR injury. First, only a minority of respondents use pharmacological agents to modify liver IR injury during hepatectomy with 6 (8%) of 72 respondents stating that they used this strategy (Figure 1). The influence of the phrasing of the question on the answer is seen in that when asked specifically whether pharmacological agents were used to modify liver IR injury in the next question, 13 (19% of 68 respondents) replied that they used these agents selectively in addition to the 3 (4%) who used them routinely. Nonetheless it is clear from this small study that pharmacological modification of IR injury during hepatectomy is only used by a minority of respondents. Despite the negative results of a randomised controlled trial of ischaemic preconditioning to reduce liver IR injury, this technique was used by 20 (28%) respondents.

The selection criteria for use of pharmacologic agents are interesting in that those clinicians using these agents employed them for major resections and in individuals with compromised liver parenchyma or function. This observation may be worth bearing in mind if a future randomized trial is to be considered as targeted use may be more appropriate than routine use in all liver resections. In terms of choice of pharmacological agent, it should be emphasised that the questionnaire restricted the responses and that only 18 responses were received to this question with n-acetylcysteine used without a loading dose being the most frequent choice. There is clearly no consensus on the optimum time for commencement of NAC or on the duration of use although the majority of respondents favoured a standard dose for all patients without adjustment for body weight.

The majority of respondents (56 (67% of cohort)) support a randomised trial and in view of patterns of current use it would appear that the role (if any) to be explored is in patients undergoing extended resection or with compromised functional hepatic reserve or with a small remnant liver after resection.

In summary, this is thought to be the first questionnaire survey of a population of clinicians undertaking surgery for colorectal liver metastases to investigate the use of pharmacological agents for modification of liver IR injury. Although the response rate was only 19%, the resultant population of 83 clinicians is the largest specialist group to contribute data to this question to date.

The results show that pharmacological modulation of liver IR injury is not a routine component of care after liver resection in contemporary hepatic surgery practice and that it is used routinely by only 3 (4%) respondents within LiverMetSurvey and selectively by a further 13 (19%) with the indications being major resections or in patients with compromised liver reserve.

## Appendix

### Questionnaire

Management of Hepatic Ischemia-Reperfusion Injury during Liver Resection.

(1) Do you undertake liver resection?
  (i) Yes
  (ii) No
(2) What strategies do you use to minimise IR injury during liver resection? (More than one option can be chosen)
  (i) Avoidance of inflow occlusion (Pringle manoeuvre)
  (ii) Use of pharmacological agents to modify IR injury
  (iii) Ischaemic preconditioning
  (iv) None
  (v) Other (please specify)
(3) Do you use pharmacological agents to modify IR injury during liver resection?
  (i) Routinely
  (ii) Never
  (iii) Selectively
(4) If you use pharmacological agents selectively, what are your selection criteria? (Please select as many options as required)
  (i) Major liver resections (3 or more Couinaud segments)
  (ii) Prolonged period of inflow occlusion
  (iii) Requirement for total vascular occlusion
  (iv) Cirrhosis
  (v) Small volume future liver remnant (FLR)
  (vi) Prior chemotherapy
  (vii) If there is evidence of deteriorating liver function in the post-operative period
  (viii) Other (please specify)
(5) If you use pharmacological agents, what do you use?
  (i) N-acetylcysteine
  (ii) Glutamine
  (iii) Other (please specify)
(6) Do you use a loading dose?
  (i) Yes
  (ii) No
(7) At which stage do you give the pharmacological agent?
  (i) Pre-operatively
  (ii) At induction of anaesthesia
  (iii) During liver mobilisation
  (iv) At commencement of liver transection
  (v) At completion of liver transection
  (vi) Post operatively (routinely)
  (vii) Post operatively if there is evidence of deterioration in liver function
  (viii) Other (please specify)
(8) If you use N-acetylcysteine, what dose do you use?
  (i) A standard dose for all
  (ii) A weight related dose
(9) If you use N-acetylcysteine infusion, how long do you continue the infusion?
  (i) 24 hours
  (ii) 48 hours
  (iii) 72 hours
  (iv) Until serum transaminases start to fall
  (v) Until normalisation of serum transaminases
(10) Do you think there should be a randomised controlled trial to evaluate the use of N-acetylcysteine in liver resection?
  (i) Yes
  (ii) No
  (iii) Do not know
